# Comparison of Concomitant Mesalamine and Immunomodulator Therapy and Immunomodulator Monotherapy for Crohn's Disease

**DOI:** 10.1155/2018/4826973

**Published:** 2018-01-18

**Authors:** Min Seob Kwak, Kyung-Jo Kim, Jae Hee Cheon, Wan Soo Kim, Jeong-Mi Lee, Sung Wook Hwang, Sang Hyoung Park, Dong-Hoon Yang, Byong Duk Ye, Jeong-Sik Byeon, Seung-Jae Myung, Suk-Kyun Yang

**Affiliations:** ^1^Department of Internal Medicine, Kyung Hee University Hospital at Gang Dong, Kyung Hee University College of Medicine, Seoul, Republic of Korea; ^2^Department of Internal Medicine, Graduate School, Yonsei University College of Medicine, Seoul, Republic of Korea; ^3^Department of Gastroenterology, Asan Medical Center, University of Ulsan College of Medicine, Seoul, Republic of Korea

## Abstract

**Background:**

Although immunomodulators are increasingly used in Crohn's disease (CD), a significant number of gastroenterologists still use 5-aminosalicylate (5-ASA) in combination with azathioprine (AZA) or 6-mercaptopurine (6-MP); there is limited evidence regarding the benefit of concomitant 5-ASA with AZA/6-MP compared with AZA/6-MP monotherapy for the treatment of CD.

**Study Design:**

A total of 106 patients who received AZA/6-MP for more than 3 months between January 1991 and May 2014 were identified retrospectively. Each patient was matched with 3 randomly selected controls who were treated with concomitant therapy during the same period.

**Results:**

The cumulative probabilities of steroid use at 5 and 10 years were 24.9% and 75.8% in the 5-ASA + AZA/6-MP group and 31.2% and 87.8% in the AZA/6-MP group, respectively (*P* = 0.187). The cumulative probabilities of anti-TNF use, resectional surgery, and disease-related hospitalization were comparable between the groups. The younger age and the use of lower doses of immunomodulators were associated with higher requirement of rescue therapy.

**Conclusions:**

This study did not demonstrate that the concomitant use of 5-ASA with AZA/6-MP showed the proof or effect in terms of steroid requirements, anti-TNF use, resectional surgery, or disease-related hospitalization compared with that of AZA/6-MP alone.

## 1. Introduction

Crohn's disease (CD) is a chronic inflammatory bowel disease of unknown etiology that leads to stricturing or penetrating complications in the gut wall [1, 2]. Traditional management of active CD includes 5-aminosalicylic acid (5-ASA), corticosteroid, and immunomodulators [3]. Anti-TNF agents have recently been added to the management of CD. The use of azathioprine (AZA) or 6-mercaptopurine (6-MP) for CD treatment is increasing [4, 5]. However, there is conflicting evidence regarding the efficacy of 5-ASA in active CD [6] and also striking discrepancies between the views of both experts and community providers regarding 5-ASA therapy for CD patients [7]. In a survey from Australia, more than half of the clinicians used AZA/6-MP concomitantly with 5-ASA [8].

There have been limited studies on concurrent therapies with 5-ASA and immunomodulators and the outcomes of these treatments in the inflammatory bowel disease (IBD) population [9, 10]. Notably, these reports had insufficient evidence to conclude that concomitant treatment with 5-ASA and immunomodulators had additional benefits over immunomodulator monotherapy for disease control in patients with CD. Thus, we performed this study to compare treatment efficacy in terms of the requirement for corticosteroids or anti-TNF agents, resectional surgery, and disease-related hospitalization in CD patients receiving concurrent therapy with 5-ASA and AZA/6-MP and those receiving AZA/6-MP monotherapy.

## 2. Patients and Methods

### 2.1. Patients

A total of 1192 CD patients who received AZA or 6-MP between January 1991 and June 2014 were identified from the IBD registry at Asan Medical Center, Seoul, Korea. A uniform definition by previously established international criteria based on clinical, endoscopic, histopathological, and radiological findings was used to diagnose CD patients [11]. After excluding patients treated with AZA/6-MP for less than 3 months or followed for less than 6 months, 1067 patients were identified. Of the 1067 CD patients, all CD patients treated with AZA/6-MP alone (*n* = 106) were identified. To perform a matched case-control study, for each AZA/6-MP monotherapy patient, we randomly selected 1 to 3 controls (*n* = 318) treated with concomitant 5-ASA + AZA/6-MP therapy from the same registry (*n* = 961). Controls were individually matched by the first visit to the Asan Medical Center due to symptoms or signs of IBD (±2 years) and calendar year at diagnosis (±2 years).

Medical records were retrospectively reviewed for patients' demographics, disease characteristics, medications, and information on resectional surgeries. Patient demographics included age and year at diagnosis, gender, smoking status at diagnosis, and whether they were lost to follow-up. Disease localization and CD behavior were described according to the Montreal classification [12]. Perianal disease was defined if specific therapy was required (abscess drainage, insertion of setons, or antibiotic/drug therapy). Information on medication use was collected, including corticosteroid (prednisolone or methylprednisolone) therapy and time to commencement. Details on immunomodulator therapy (including AZA/6-MP) included time to commencement, maximum dose tolerated, and duration.

The Institutional Review Board of the Asan Medical Center approved this study (IRB number 2014-1083).

### 2.2. Treatment Strategies and Follow-Up Protocol

Oral aminosalicylates were used to induce and maintain remission in patients with mild to moderately active disease until the mid 2000s [2]. AZA/6-MP was used as maintenance therapy for steroid-dependent, steroid-refractory, or fistulizing patients in selected cases, mainly after bowel resectional surgery, but on a more widespread basis from the mid to late 2000s. To minimize the occurrence of unexpected severe leucopenia, AZA was started at 25–50 mg/day and then increased by 25–50 mg/day every 2–4 weeks to a maximum dose of 2.0–2.5 mg/kg/day while leukocyte levels were monitored. If a rapid decrease in the leukocyte count or leucopenia occurred, the dose of AZA was decreased or discontinued temporarily and then restarted at a lower dose. The dose of AZA was defined as the maintenance dose that induced remission.

6-MP dose was converted to an equivalent AZA dose by multiplying by 2.08 [13]. Systemic corticosteroid (oral prednisone, 40–60 mg/day) was given to patients with moderate to severe disease or to treat flares, then tapered and discontinued over 2-3 months. Anti-TNF agents were administered from the early 2000s to patients with moderate to severely active disease who failed or were intolerant to corticosteroids and/or AZA/6-MP. Bowel resectional surgery was performed to treat medical refractory disease or complicated diseases such as intestinal obstruction, perforation, and abscess [2].

### 2.3. Outcome Measures

The primary outcome measure for this study was the requirement for rescue therapy, such as corticosteroid or anti-TNF agents, at each point during the study period. The secondary outcomes were the cumulative probabilities of intestinal resectional surgery and disease-related hospitalization and predictive factors associated with rescue therapy in CD patients.

### 2.4. Statistics

Data analyses were conducted using SPSS software, version 21.0 (SPSS Inc., Chicago, IL). A two-tailed Student's *t*-test was used for continuous variables, and a two-tailed *χ*^2^ test or Fisher's exact test was used for categorical data. Long-term outcomes were plotted using the Kaplan-Meier survival method and a log-rank test for grouped factors over the observational period. The joint effect of various risk factors on the primary outcomes was assessed using multivariate Cox regression analysis. Two approaches were used to assess the validity of the proportional hazards assumption. First, the assumption was assessed by log-minus-log survival function and found to hold. Second, to confirm the assumption of proportionality, time-dependent covariate analysis was used. The time-dependent covariate was not statistically significant, suggesting that the proportional hazards assumption is reasonable. Estimates for hazard ratios (HR) and 95% confidence intervals (CI) were calculated from these regression models. Statistical significance was defined as a *P* value < 0.05.

## 3. Results

### 3.1. Baseline Patient Characteristics

Demographic data and clinical characteristics are listed in Table 1. There were no significant differences between the two groups in terms of age, gender, location of disease, and behavior. There was also no difference in past steroid use (*P* = 0.072). The rate of previous intestinal resection was not different between the two groups (*P* = 0.130). However, the maximal tolerable dose of AZA/6-MP was higher in the AZA/6-MP-only group compared with the 5-ASA + AZA/6-MP group (*P* < 0.001). Most of the patients received immunomodulators after the year 2005, 93.4% (99/106) in the AZA/6-MP group and 88.1% (280/318) in the 5-ASA + AZA/6-MP group (*P* = 0.122).

The median follow-up duration after diagnosis (57.6 months; range, 192.0 months; interquartile range, 28.8 months) was identified in both groups. And, no patients with colorectal cancer or dysplasia were identified in this study.

### 3.2. Requirement of Rescue Therapy

**Figure 1:**
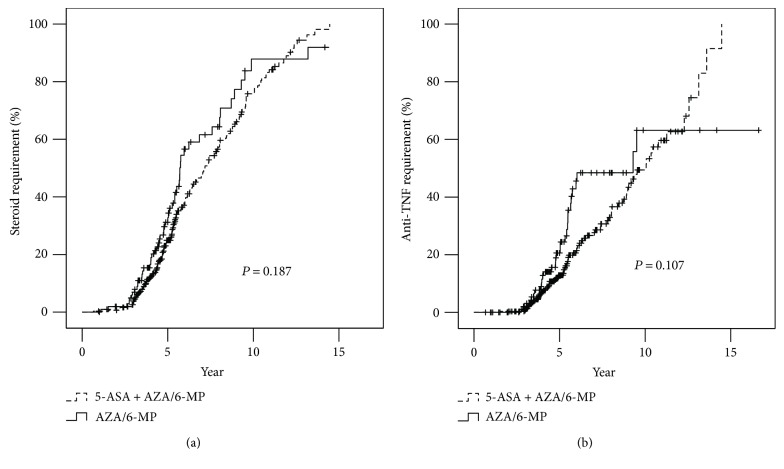
Cumulative probabilities of patients requiring steroids (a) and biological agent treatment (b).

During follow-up, corticosteroids were used at any time in 49.1% of the AZA/6-MP group and 49.4% of the 5-ASA + AZA/6MP group. The cumulative probabilities of a requirement for corticosteroid at 5 and 10 years were 24.9% and 75.8% in the 5-ASA+AZA/6-MP group and 31.2% and 87.8% in the AZA/6-MP group, respectively. No statistically significant difference was found between the two groups (log-rank test, *P* = 0.187; Figure 1(a)).

Anti-TNF agents were used in 32.1% of the AZA/6-MP group and 26.1% of the 5-ASA + AZA/6-MP group. The cumulative probabilities of anti-TNF agent use at 5 and 10 years were 12.9% and 49.4% in the AZA/6-MP group and 20.6% and 63.2% in the 5-ASA + AZA/6-MP group, respectively. No statistically significant difference was found between the two groups (log-rank test, *P* = 0.107; Figure 1(b)).

### 3.3. Intestinal Resectional Surgery and Disease-Related Hospitalization

**Figure 2:**
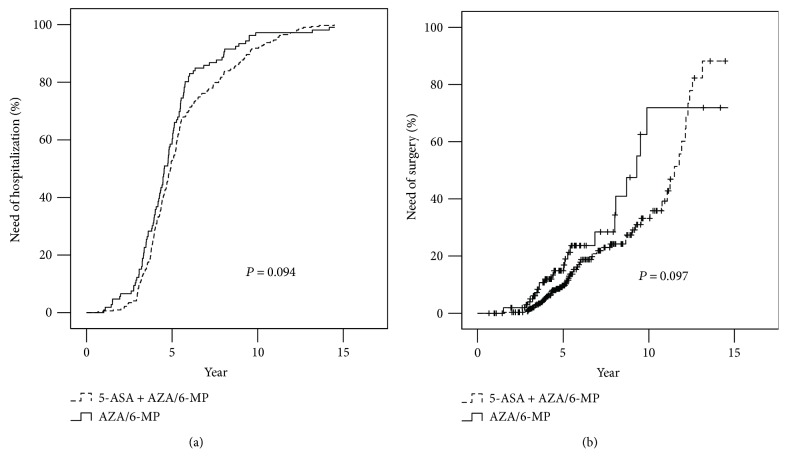
Cumulative probabilities of patients requiring hospitalization (a) and surgery (b).

Eighty-three (19.6%) of the patients underwent intestinal resectional surgery. The cumulative probabilities of intestinal resection at 5 years and 10 years were 6.2% and 33.2% in the 5-ASA + AZA/6-MP group and 14.9% and 71.9% in the AZA/6-MP group, respectively (Figure 2(a)). No statistically significant difference was found between the two groups (log-rank test, *P* = 0.097; Figure 2(a)).

The cumulative probabilities of disease-related hospitalization between the two groups were not significantly different at 5 and 10 years (52.8% and 91.8% in the 5-ASA + AZA/6-MP and 59.4% and 95.3% in the AZA/6-MP group, resp.; log-rank test, *P* = 0.094; Figure 2(b)).

### 3.4. Factors Associated with Rescue Therapy

The only variables that were associated with steroid and anti-TNF agent rescue therapy were age and maximal tolerable dose of immunomodulators. The younger age and the use of lower doses of immunomodulators were associated with higher requirement of rescue therapy (steroid rescue therapy: HR = 0.959, 95% CI = 0.938 to 0.980 and HR = 0.548, 95% CI = 0.315 to 0.953; anti-TNF rescue therapy: HR = 0.958, 95% CI = 0.930 to 0.987 and HR = 0.385, 95% CI = 0.145 to 1.025) (Table 2). Although gender for anti-TNF rescue therapy and previous intestinal resection for steroid rescue therapy were of statistical significances, the factors were not associated with the other rescue therapies (Table 2). There were no associations of previous steroid use with both steroid and anti-TNF rescue therapies (*P* = 0.079, *P* = 0.604).

### 3.5. Economic Issues

The total annual cost to treat a patient with 60 kg in Korea is approximately 210 USD for AZA/6-MP monotherapy (AZA, 2 mg/kg) and approximately 907 USD for 5-ASA + AZA/6-MP (AZA, 2 mg/kg).

## 4. Discussion and Conclusions

In our present study, we found that coadministration of 5-ASA and AZA/6-MP was not more effective than AZA/6-MP alone in terms of the requirement for rescue medications such as steroids and anti-TNF agents. Furthermore, the cumulative probabilities of hospitalization and intestinal resectional surgery were similar between the groups of patients on either regimen.

Recently, several studies have demonstrated that the early and widespread use of AZA/6-MP reduces surgical rates in clinical practice, which has led to increased administration of AZA/6-MP. At our center, AZA/6-MP has been used earlier and more frequently over the past 30 years [2, 4]. AZA/6-MP is also used as maintenance treatment for moderate to severe CD after corticosteroid induction or concomitant with anti-TNF agents [14–17].

As far as we know, there have only been two studies that have attempted to determine if concurrent therapy with 5-ASA + AZA/6-MP improved disease control in IBD patients [9, 10]. Of these two reports, only the retrospective UK study included CD patients in their calculation of remission and compared relapse rates [10], and the authors concluded that concurrent use of 5-ASA drugs with immunomodulators did not reduce the relapse rates of IBD patients who were established on azathioprine therapy. However, methodological problems make it difficult to draw a definite conclusion from these earlier data.

In this study, we calculated the cumulative probability of corticosteroid and anti-TNF agent use during about 5 years of follow-up in AZA-only and 5-ASA + AZA/6-MP groups. We demonstrated in our analyses that there were no significant differences in rescue medication, hospitalization, or resectional surgery between these two groups. In clinical practice, steroid use, anti-TNF introduction, and surgical intervention might be useful surrogate markers for assessing disease severity and flare.

There are some possible explanations for no more efficacy in the 5-ASA + AZA/6-MP group than in the AZA/6-MP group. One reason might be attributed to that the patients in the concomitant therapy group were given lower doses of AZA/6-MP than those in the monotherapy group. The other explanation was that 5-ASA has a limited beneficial effect on management of CD.

There have been case reports of leukopenia occurring after the addition of 5-ASAs to thiopurines, although the clinical effect of this interaction is likely small [18]. Furthermore, there have been reported some warnings about adverse events caused by drug-drug interaction between AZA/6-MP and 5-ASA [19–21]. Finally, in choosing a treatment, cost-effectiveness needs to be considered as it is associated with patient compliance. Cost is also an important issue for treating patients with CD, as long-term treatment is essential. It is well-known that 5-ASA therapy is not expensive [22]. When considering the cost of drugs itself, we determined that the cost per patient might up to 4.5 times the annual cost of AZA/6-MP (907 USD versus 210 USD).

None of the patients with colorectal cancer and dysplasia were identified. Relatively short duration of a follow-up period and small number of the patients could be the reason of no occurrence of colorectal cancer or dysplasia in both groups.

Several previous studies demonstrated that steroid use [23], young age [23, 24], and low dose of immunomodulators [25, 26] were associated with lack of response or disabling disease in CD patients, but little is known about clinically predictive factors for further rescue therapy during immunomodulator therapy. In the present study, age and maximal tolerable dose of immunomodulators were negatively associated with use of rescue therapy. This result might be explained by that we should pay attention to young CD patients in prescribing immunomodulators.

Taken together, based on these findings, we did not find the proof of effect that concomitant use of 5-ASA on AZA/6-MP may be effective and may be associated with higher costs.

This study had several limitations of note. First, its retrospective design might have induced selection and information-gathering biases. For example, it is possible since we only enrolled the patients who were tolerant to AZA/6-MP. And we did not gather the frequency of adverse events because we enrolled relatively well-tolerant patients. Second, our study did not include specific information about the disease severity, active disease control, or endoscopic findings because of its retrospective design. Thus, we could not demonstrate the rate of mucosal healing. Instead, we demonstrated the requirement of rescue medication and hospitalization and requirement of resection surgery, which might be useful endpoints. Third, median follow-up in our patient series was approximately 57 months, which was not long enough to demonstrate the occurrence of colorectal cancer. Fourth, since this study was conducted retrospectively, the unmeasured or missed confounders, such as various formulations and detailed dosages of mesalamine, the presence of smoking history, or extraintestinal manifestation, may affect the outcome. Fifth, we did not conduct to assess the quality of life of the patients during treatment, which may influence the clinical outcome. Finally, the present study could not have enough power to detect our 6% difference between the groups because of small sample size. So, prospective, large-scale, multicenter studies for CD patients are needed to assess and compare long-term outcomes of the AZA-only group with those of the 5-ASA + AZA/6-MP groups more objectively and precisely.

In conclusion, concurrent 5-ASA and AZA/6-MP therapy may not be more effective than AZA/6-MP monotherapy in terms of the requirements for rescue therapy, hospitalization, and resectional surgery in CD patients.

## Figures and Tables

**Table 1:** Characteristics of the patients.

Characteristics	AZA/6-MP (*n* = 106)	AZA/6-MP with 5-ASA (*n* = 318)	*P* value
Age, yr, median (IQR)	30.0 (8.0)	31.0 (11.0)	0.290
Gender (%)			0.844
Male	81 (76.4)	240 (75.5)	
Female	25 (23.6)	78 (24.5)	
Location (%)			0.558
L1 (ileal)	11 (10.4)	42 (13.3)	
L2 (colonic)	4 (3.8)	16 (5.0)	
L3 (ileocolonic)	56 (52.8)	171 (53.8)	
L4 (only upper GI)	0 (0.0)	0 (0.0)	
L1–L4	6 (5.7)	21 (6.6)	
L2–L4	2 (1.9)	1 (0.3)	
L3-L4	25 (23.6)	64 (20.1)	
NA	2 (1.8)	3 (0.9)	
Behavior at diagnosis (%)			0.781
B1 (nonstricturing, nonpenetrating)	21 (19.9)	71 (22.3)	
B2 (stricturing)	10 (9.4)	28 (8.8)	
B3 (penetrating)	21 (19.9)	45 (14.2)	
B1-P	27 (26.4)	87 (27.4)	
B2-P	10 (9.4)	27 (8.5)	
B3-P	16 (15.1)	57 (17.9)	
NA	1 (0.9)	3 (0.9)	
Previous intestinal resection (%)	5 (4.7)	5 (1.6)	0.130
Start year of immunomodulators (%)			0.122
1991–2005	7 (6.6)	38 (11.9)	
2006–2014	99 (93.4)	280 (88.1)	
Previous steroid use (%)	23 (21.7)	98 (30.8)	0.072
AZA/6-MP max. tolerable dose, median (IQR)	1.7 (1.5)	1.2 (0.8)	<0.001
Duration of AZA/6-MP use, mo, median (IQR)	49.8 (32.0)	54.6 (31.0)	0.407

AZA: azathioprine; 6-MP: 6-mercaptopurine; IQR: interquartile range; GI: gastrointestinal; NA: not applicable.

**Table 2:** Cox regression analysis for rescue therapy adjusting for various prognostic factors.

Variables	HR (95% CI)	*P* value
*Steroid rescue therapy*		
Age	0.959 (0.938–0.980)	<0.001
Gender		
Female	1.315 (0.949–1.824)	0.100
Male		
Previous intestinal resection		
Yes	3.626 (1.650–7.969)	0.001
No		
Previous steroid use		
Yes	1.040 (0.616–1.755)	0.079
No		
AZA/6-MP max. tolerable dose	0.548 (0.315–0.953)	0.033

*Anti-TNF rescue therapy*		
Age	0.958 (0.930–0.987)	0.005
Gender		
Female	1.575 (1.027–2.415)	0.037
Male		
Previous intestinal resection		
Yes	0.793 (0.108–5.817)	0.820
No		
Previous steroid use		
Yes	1.111 (0.746–1.656)	0.604
No		
AZA/6-MP max. tolerable dose	0.385 (0.145–1.025)	0.046

AZA: azathioprine; 6-MP: 6-mercaptopurine; HR: hazard ratio; CI: confidence interval.
